# Intrinsic and Extrinsic Factors Simultaneously Modulated the Use of Roadways by Golden Eagles During Winter

**DOI:** 10.1002/ece3.71955

**Published:** 2025-08-14

**Authors:** Joshua F. Layfield, Bryan Bedrosian, Anna Chalfoun, Robert Domenech, Stephen B. Lewis, Brian W. Smith, Jerod A. Merkle

**Affiliations:** ^1^ Wyoming Cooperative Fish and Wildlife Research Unit Laramie Wyoming USA; ^2^ Teton Raptor Center Wilson Wyoming USA; ^3^ Department of Zoology and Physiology University of Wyoming Laramie Wyoming USA; ^4^ Cooperative Fish and Wildlife Research Unit USGS Wyoming Laramie Wyoming USA; ^5^ Raptor View Research Institute Missoula Montana USA; ^6^ U.S. Fish and Wildlife Service Alaska Juneau USA; ^7^ U.S. Fish and Wildlife Service East Lansing Michigan USA

**Keywords:** extrinsic, habitat selection, home range, intrinsic, risk–reward, road ecology, satellite telemetry, winter range, Wyoming

## Abstract

Animals often face trade‐offs wherein foraging opportunities may coincide spatiotemporally with higher risk of mortality, especially within landscapes altered by humans. Resolving such trade‐offs can depend on intrinsic factors such as age and sex, and extrinsic factors (e.g., resource availability) within the surrounding environment. Yet both are rarely assessed simultaneously. Roads constitute a widespread form of human‐induced habitat alteration that concurrently offer potential food rewards, in the form of carrion from roadkill, and risk in the form of death by vehicle collision. We evaluated the extent to which suites of intrinsic and extrinsic factors modulated the selection for areas near roads by a large opportunistic scavenger, the golden eagle ( *Aquila chrysaetos* ). We used a collaborative, multi‐agency telemetry dataset of 51 golden eagles overwintering in Wyoming, USA during 2014 to 2023 representing 175 unique eagle‐years. Both intrinsic and extrinsic factors influenced road use. Males were 14.2 times more likely than females to select areas closer to roads. Adults, compared with their subadult counterparts (Subadult I, Subadult II, and Subadult III) were 16.3, 16, and 15.3 times more likely to select areas near roads, respectively. Moreover, individuals were more likely to select areas near roads during periods with higher snow depth on the landscape. Specifically, during periods of high snow depth (> 17.9 cm) golden eagles were 17 times more likely to select areas near roads than during periods with no snow. Our results suggest that both intrinsic and extrinsic factors influenced proximate habitat decisions and the use of areas near risky landscape elements. Our work has important implications for the contexts under which efforts to reduce golden eagle mortality, such as the removal of carcasses from roads, would be most effective.

## Graphical abstract

Golden eagles in the western United States are increasingly vulnerable to anthropogenic threats such as motor‐vehicle collisions, particularly during winter when live prey can be most limited and ungulate roadkill is more prevalent on the landscape. However, we lack an understanding of how other factors pertinent to eagles during winter (i.e., relative prey availability, relative roadkill densities, snow depth, and timing of winter) and intrinsic factors (i.e., sex, age, and migration strategies) may influence the selection of areas near roads. Using a collaborative dataset of GPS‐telemetered golden eagles (*n* = 51), we tested multiple hypotheses on how intrinsic and extrinsic factors may influence the selection of areas near roads.

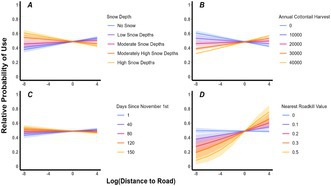

## 1 Introduction

The spatiotemporal distribution of resources or factors extrinsic to an individual (e.g., food) and risk (e.g., areas of high predator activity) can shape behavioral decisions made by animals such as habitat selection (Schmitt and Holbrook 1985), residency time (Courant and Fortin 2012), home range formation (Ullmann et al. 2018) and movement strategies (Mueller and Fagan 2008). In some cases, however, habitats with better resources can coincide with areas with higher risk, creating trade‐offs that animals must constantly balance when making spatial choices (Johnson et al. 2015; Brown and Kotler 2004). For example, female Caribou ( *Rangifer tarandus* ) adjusted their antipredator response based on the proximity to wolves ( *Canis lupus* ) and areas with relatively abundant forage (Basille et al. 2015). Additionally, factors that are intrinsic to individuals are emerging as central in modulating the spatial decisions of animals (Malagnino et al. 2021). Variation in age (Louhi et al. 2014; Poessel et al. 2016), sex (Paton et al. 2017), or migration strategy (Nicholson et al. 1997), for example, can explain relatively large amounts of variation in habitat selection. Moreover, elements of human‐induced rapid environmental change, such as roads or agricultural lands have altered risk–reward landscapes, creating novel external rewards and risks that animals must navigate (Frid and Dill 2002; Sih et al. 2011). For instance, the availability of agricultural forage in unprotected areas adjacent to protected areas has created novel trade‐offs between agricultural food subsidies and risk of harvest by humans for some species (Sigaud et al. 2017).

Whereas both intrinsic (Foster et al. 2010; Wat et al. 2020) and extrinsic (Guest et al. 2022; Moreira et al. 2018) factors can influence the use of risky landscape features, few studies focus on their impact simultaneously. Without simultaneous assessments, context‐specific responses could be obscured, making it difficult to disentangle the mechanisms modulating responses to human‐induced habitat changes (Moore et al. 2023). For instance, the extent of site fidelity can be related to the distribution of high‐quality habitats, an intrinsic preference for the locations based on familiarity, or a combination of both (Picardi et al. 2022; Piper 2011). Accounting for intrinsic factors of individuals can therefore clarify the strength of the effect of extrinsic factors of interest (Picardi et al. 2022). Low food availability, for example, might disproportionately affect younger individuals because they have less experience foraging or hunting, which could result in more risk‐prone behavior such as the use of anthropogenic structures. Behaviors and choices also can vary by sex. For example, adult female golden‐mantled ground squirrels (*Callospermophilus lateralis*) in Colorado, USA, were more susceptible to motor‐*vehicle* collisions than males, likely because of their territorial behaviors during peak traffic seasons (Burgstahler et al. 2023).

The public road network is one of the most prominent elements of human‐induced rapid environmental change on Earth. Roads and vehicular traffic impact nearly 20% of the land area in the United States (Forman and Alexander 1998); another 25 million km of new roads are expected to be built worldwide by 2050 (Laurance et al. 2014). Roads contribute significantly to habitat loss, fragmentation, and direct mortality. Each year, 1–2 million vehicle collisions with wild mammals (van der Ree et al. 2011) and approximately 200 million with birds (Loss et al. 2015) occur in the United States. Yet, roads can also provide beneficial foraging opportunities. For example, forest ravens ( *Corvus tasmanicus* ) have potentially expanded their range due to greater densities of roadkill and natural carrion in parts of Tasmania (Cunningham et al. 2018; Fielding et al. 2020). Roadside embankments and ditches can provide high‐quality and/or seasonally accessible forage for herbivores (Hill et al. 2021). Nonetheless, the benefits of roads do not always outweigh the consequences. In some contexts, individuals can experience relatively high rates of mortality from motor‐vehicle collisions while attempting to forage on roadkill (Phillips 1986).

Our objective was to disentangle the importance of both intrinsic and extrinsic factors in modulating the use of anthropogenic features that simultaneously can enhance foraging opportunities yet increase the risk of mortality. We focused our work on a large, migratory, diurnal raptor, the golden eagle ( *Aquila chrysaetos* ). Golden eagles are long‐lived (≤ 30 years, Harmata 2012) partially migrant species, with low annual reproductive potential (Millsap et al. 2022). Leporids tend to be the primary prey for golden eagles in the western USA during both breeding and non‐breeding periods (Bedrosian et al. 2017; Preston et al. 2017). Leporid populations tend to fluctuate dramatically (and cyclically) over time (Bartel et al. 2008; Fedy and Doherty 2011). Notably, golden eagles also forage on roadkill, particularly during the non‐breeding period (Bedrosian et al. 2017; Slater et al. 2022), making them increasingly vulnerable to mortality from motor vehicle collisions (Lonsdorf et al. 2018; Slater et al. 2022). In one non‐breeding period in southwest Wyoming, USA, nearly 100 eagle mortalities were documented along a single secondary road (Phillips 1986).

We first assessed the extent to which golden eagles generally selected areas near roads during winter. Second, we evaluated alternative hypotheses for which intrinsic factors (age, sex, and migratory strategy) most influenced the selection of areas near roads (see Tables 1 and 2, and the Section 2 for details of the predictions and the statistical framework for each intrinsic and extrinsic hypothesis). Finally, while accounting for influential intrinsic factors, we evaluated hypotheses related to how extrinsic factors known to influence foraging success (snow depth, an index of roadkill availability, time of season, and an index of primary prey availability) modulated the selection of areas near roads (Table 2; Kostrzewa and Kostrzewa 1991; Oro and Furness 2002; Slater et al. 2022; Sonerud 1986). We focused on the non‐breeding period (winter) because most wildlife vehicle collisions in our study area are reported during the fall and winter (Riginos et al. 2016; Roy and Ksaibati 2021), and the likelihood for eagle‐vehicle collisions is highest (Lonsdorf et al. 2018). Moreover, the demography and behavior of golden eagles' primary and secondary prey species suggest that they are most limiting during winter (Gross et al. 1974; Harlow and Menkens Jr. 1986; Hayden 1966). Finally, studying during winter facilitated the opportunity to compare behaviors between year‐round and migratory individuals.

**TABLE 1:** Generalized linear mixed‐model structures of intrinsic hypotheses and their predictions for golden eagle selection for areas near roads during winter at the home range scale from 2014 to 2023 in Wyoming, USA.

Hypothesis	Model name	Model structure	Prediction
*H*_base_	Just distance to roads	Used locations ~ log(D2R) + Autocov + (1|AID‐winter)	
H1	Sex‐dependent variation	Used locations ~ log(D2R) + log(D2R): Sex + Autocov + (1|AID‐winter)	Selection is best explained by males
H2	Age‐dependent variation	Used locations ~ log(D2R) + log(D2R): Age + Autocov + (1|AID‐winter)	Selection is best explained by sub‐adults
H3	Sex‐ & age‐dependent variation	Used locations ~ log(D2R) + log(D2R): Sex + log(D2R): Age + log(D2R): Age:Sex + Autocov + (1|AID‐winter)	Selection is best explained by male sub‐adults
H4	Movement strategy	Used locations ~ log(D2R) + log(D2R): MovementStrategy + Autocov + (1|AID‐winter)	Selection is best explained by residents
*H*_Intrinsic Global_	Global model	Used locations ~ log(D2R) + log(D2R): Sex + log(D2R): Age + log(D2R): MovementStrategy + log(D2R): Age:Sex + Autocov + (1|AID‐winter)^6^	Selection is best explained by individuals that are male, subadult, and residents

*Note:* The values in the “Prediction” column indicate the traits we predicted to be more associated with the selection of areas near roads. (D2R) denotes ‘distance to road’. (Autocov) represents the spatial autocovariate to account for spatial autocorrelation. (AID) represents an animal's identification. *H*_base_ is the base model, which includes the log (D2R) and random intercept, and H5 is the global model.

**TABLE 2:** Generalized linear mixed‐model structures of extrinsic hypotheses and their predictions for golden eagle selection for areas closer to roads during winter in Wyoming, USA, at the home range scale during 2014–2023.

Hypothesis	Model name	Model structure	Prediction
H5	Roadkill availability	Used locations ~ Top Intrinstic Model + log(D2R):Nearest_Roadkill_Density + Autocov + (1|AID‐winter)^2^	Selection is best explained by roadkill hotspots
H6	Prey constraint (Snow cover)	Used locations ~ Top Intrinstic Model + log(D2R): Snow_Depth + Autocov + 1|AID‐winter)	Selection increases with snow depth
H7	Prey energetic constraints	Used locations ~ Top Intrinstic Model + log(D2R): Time_Across_Winter + Autocov + (1|AID‐winter)	Selection decreases over time
H8	Diminishing prey availability	Used locations ~ Top Intrinstic Model + log(D2R): Time_Across_Winter + Autocov + (1|AID‐winter)	Selection increases over time
H9	Primary prey availability	Used locations~ Top Intrinstic Model + log(D2R): CottontailHarvest + Autocov + (1|AID‐winter)	Selection increases in years with lower rabbit harvest

*Note:* Model structures for the extrinsic hypothesis suite included the fixed effects from the top‐performing intrinsic model, the variable of interest associated with the pertinent extrinsic hypothesis, and AID‐winter to account for individual variation across years. (D2R) denotes distance to road. (Autocov) represents the spatial autocovariate to account for spatial autocorrelation.

## 2 Materials and Methods

### 2.1 Study Area

Our study took place across the state of Wyoming, USA. Wyoming hosts some of the highest densities of breeding golden eagles in the contiguous United States (Nielson et al. 2014), and provides important habitat for resident birds and for influxes of migratory individuals during winter (Bedrosian et al. 2018; Nielson et al. 2017; Wallace et al. 2024). During winter, golden eagles tend to use areas within the intermountain basins of the western and central parts of the state, and/or grasslands with dispersed croplands in the east (Stahl and Curran 2017; Wallace et al. 2024). The state is roughly 250,000 km^2^ with elevations ranging from 1000 to 4200 m (Driese et al. 1997). Temperatures and precipitation are variable. From 1991 to 2020, the average minimum temperatures in Wyoming during winter ranged from −15°C to −8°C with lower temperatures at higher elevations (Frankson et al. 2022). Per estimated annual snowfall from 1991 to 2020, lower elevations (e.g., Boysen Dam State Park) can receive ≤ 40 cm (cm) of snow per year, whereas higher elevations in montane regions (e.g., Lake Village, Yellowstone National Park) can receive ≥ 500 cm (Palecki et al. 2021). During 2010–2019, Wyoming reported roughly 27,000 wildlife‐vehicle collisions, and collisions peaked during late fall/early winter (Roy and Ksaibati 2021). More than 80% of those collisions involved large ungulates, particularly mule deer (Roy and Ksaibati 2021).

### 2.2 Animal Capture and Data Preparation

We leveraged a large Global Positioning System (GPS) based dataset on telemetered golden eagles collected previously by numerous collaborators from state, federal, and non‐governmental organizations. Animals were captured in Wyoming, Montana, Alaska, Colorado, and Nebraska for independent study objectives (Federal Permit #MB24140 & #MB21678; MT State Permit #Year‐070‐W; State of AK Permit 16‐020; USFWS IACUC Protocol 2012‐013; ADFG IACUC Protocol 2013‐036). All individual golden eagles were captured using bow nets, net launchers, or were tagged in the nest before fledging. Each individual was fitted with 30, 45, or 75 g GPS transmitters (Microwave Telemetry or Geotrack Inc.), set to return hourly GPS locations from approximately sunrise to sunset throughout the day. All birds were aged by molting patterns and plumage characteristics up to adulthood (4 years of age and older, Bloom and Clark 2001) and were classified as either ‘Juvenile’ (≥ 1 year old), ‘Subadult I’ (≥ 1 year old), ‘Subadult II’ (≥ 2 year old), ‘Subadult III’ (≥ 3 year old), or ‘Adult’ (≥ 4 years old). We excluded the juvenile age class from our analyses, as juveniles can remain in their natal territory and/or still have codependency with their parents during the post‐fledging period (Hemery et al. 2023). We used physical characteristics such as toe pad length, body weight, hallux length, and a combination of head measurements and genetic analysis to determine sex (Bortolotti 1984; Edwards and Kochert 1986; Harmata and Montopoli 2013). All techniques adhered to guidelines within the Raptor Research and Management Techniques (Bird and Bildstein 2007) and Guidelines to the Use of Wild Birds in Research (Fair et al. 2010).

We defined winter as November 1st of one calendar year to March 31st of the following year, as this encompassed the wide breadth of timing for migrating golden eagles entering and leaving their winter home ranges (Domenech et al. 2015). We visually inspected each individual's GPS data and manually removed any obvious (fast and directional) migratory movements into or out of a given individual's winter range at the beginning or end of each winter (Bedrosian et al. 2018). For instance, most individuals would depart from their winter range quickly (within a given day) covering hundreds of kilometers away from their winter home range. To determine whether birds wintered in Wyoming, we first applied a 150‐km spatial buffer around the state boundary and intersected all GPS locations within the buffered polygon using the st_intersects() function within the ‘sf’ package in Program R (R Core Team 2023). Doing so accounted for individuals whose home ranges spanned state lines. We then filtered the movement data to individuals that spent at least 30 days within the buffered polygon during a given winter season, as this cutoff appears indicative of winter‐home‐range formation for golden eagles (Domenech et al. 2015). We manually inspected each individual in each year and included individuals that met the inclusion criteria above, and classified each individual as ‘resident’ or ‘migrant’ based on whether they had a non‐overlapping summer range north of 50° latitude (Wallace et al. 2024).

Our final dataset included 146,310 GPS locations from 51 individual golden eagles spanning 2014–2023, creating 175 unique animal ID winters (hereafter, ‘AID‐winters’) from 24 males and 27 females. Tallies of the age classes across the study period included 17 individuals at Subadult I, 22 individuals at Subadult II, 31 individuals at Subadult III, and 105 individuals at the adult age class. Of the 175 AID‐winters, 137 were identified as ‘Resident’ and 38 were ‘Migrant’. All golden eagles that wintered in Wyoming more than once maintained their ‘Migrator’ or ‘Resident’ classification (i.e., there were no changes in migration strategy between years).

### 2.3 Resource Selection Function Framework

**FIGURE 1:**
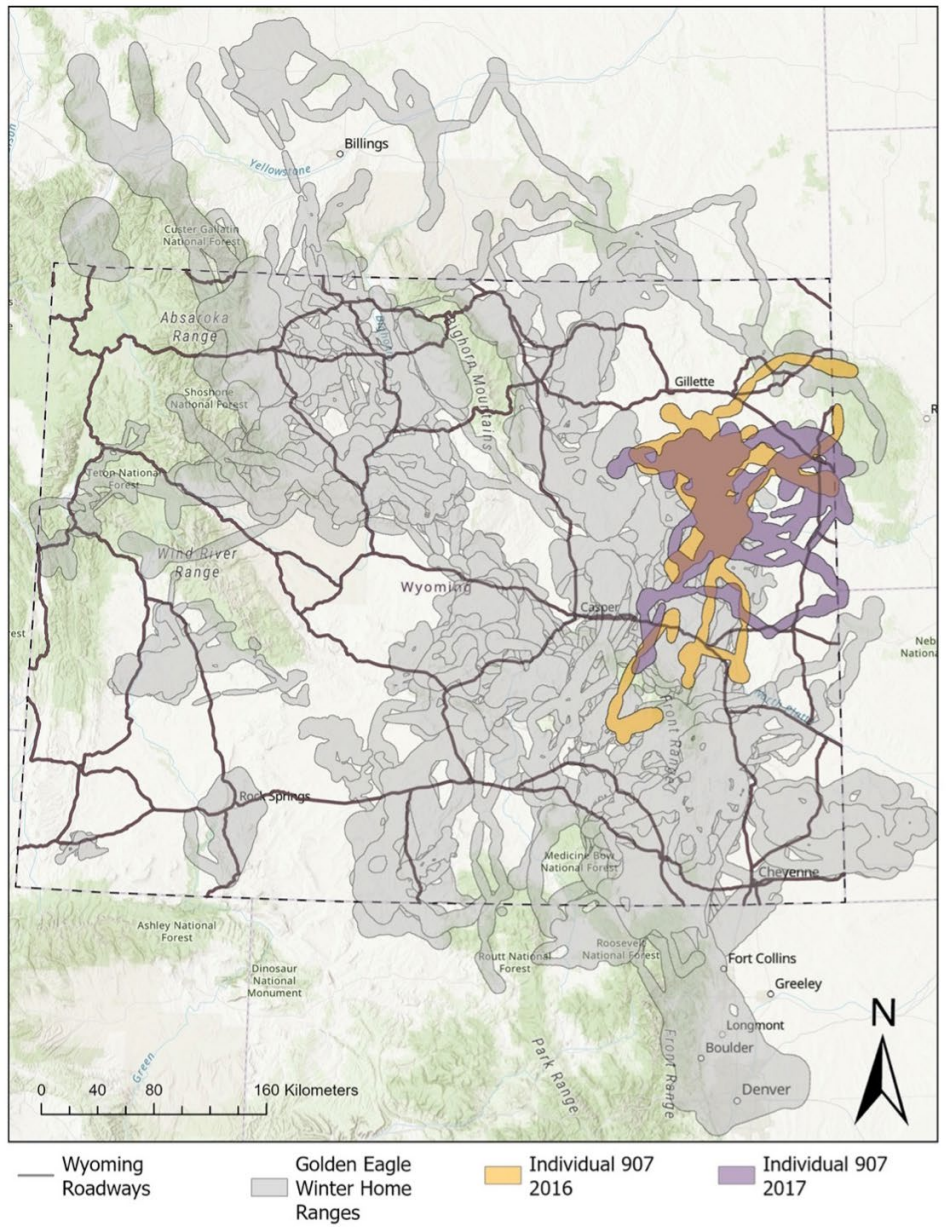
Golden eagle winter home ranges during 2014–2023 in Wyoming, USA. Each polygon (in gray) represents an individual's home range for a given winter AID‐winter. For example, individual 907's home ranges during the 2 years are highlighted in orange and purple. All home ranges represent the 99.9% contour of the occurrence distribution calculated from Brownian Bridge Movement Models.

We used a Resource Selection Function (RSF) framework to assess the importance of both intrinsic and extrinsic factors in modulating the use of areas near roads by golden eagles. RSFs estimate the relative probability of use of a resource unit by contrasting resources at observed points to available points (Boyce et al. 2002). We investigated resource selection within home ranges wherein used GPS locations for a given animal ID‐winter were compared with available locations drawn randomly from within each AID‐winter's home range at a ratio of one available location for every used location (Fattebert et al. 2015; Paolini et al. 2018; van Beest et al. 2016). To assess whether we drew enough available points to correctly quantify availability, we resampled our available points, reextracted GIS data, and refit our models. All the qualitative components of our results (significance, direction of the betas, etc.) did not change; thus, the number of available points we drew seemed to be adequate and robust (Street et al. 2021). To delineate winter home ranges, we used the 99.9% contour around the occurrence distributions calculated from Brownian Bridge Movement Models (BBMMs) applied to each individual's observed GPS data for each winter (Horne et al. 2007; Figure 1). BBMMs estimate the probability that an individual will use an area (i.e., occurrence distribution) based on the time elapsed between GPS locations, the speed of the individual, and distance traveled between GPS locations (Horne et al. 2007). Therefore, using a 99.9% contour around the occurrence distributions allowed us to capture available habitat around the area the animal used during winter (Horne et al. 2007).

We developed a grid (30‐m resolution) that represented the distance to the nearest interstate, national, or state highway (hereinafter “roads”; where most roadkill occurs) based on TIGER data (United States Census Bureau (USCB) 2016). For each used and available location, we extracted the distance to the road. Since relatively small increments of road distance are likely more important when animals are close to roads versus far, we used the natural logarithm of distance to road.

### 2.4 Intrinsic and Extrinsic Factors Modulating Use of Roads

To assess the extent to which golden eagles selected areas near roads during winter, we fit a base model with “distance to road” as the only variable (see *H*_Base_ in Table 1). We then tested intrinsic hypotheses, including sex, age, and movement strategy, by including an interaction term for each intrinsic factor with distance to road (Table 1). Because males, particularly younger sub‐adults, tend to have larger and more variable winter home ranges (Miller et al. 2017), we predicted that sub‐adult males would select more strongly for areas near roads, given that they were more likely to encounter roadways (H1 & H3 in Table 1; Miller et al. 2017). In terms of age, we predicted sub‐adults would select areas near roads more than adult age classes because of their relative inexperience and known susceptibility to, and association with, risky landscape elements (H2, Table 1; Hixson et al. 2022; Hunt et al. 1999). Finally, we predicted that residents would be more likely to select roads than migrants because of their knowledge of the spatial distribution of roadkill hotspots and experience using them before the influx of migrants in the fall (H4, Table 1; Van Moorter et al. 2009; Wallace et al. 2024).

Once we determined the top intrinsic model (see below for model selection criteria), we added another interaction term with distance to road to test each variable representing five extrinsic hypotheses in separate models (Table 2). We assessed each extrinsic variable separately to avoid convergence issues and ensure the interpretability of the models. First, we hypothesized that golden eagles would be particularly attracted to roads where the availability of potential food rewards was greatest (H5: roadkill availability). To create a spatial index of roadkill probability, we used Kernel Density Estimates of wildlife–vehicle collisions (consisting primarily of mdeer) occurring along 1.6 km segments along interstates and highways in Wyoming from 2008 to 2013 (Riginos et al. 2016). For each used and available location, we extracted the roadkill density value of the nearest cell in the roadkill density layer.

Our second extrinsic hypothesis (H6: prey constraint, snow cover) was that deeper snow could hamper the locomotion or activity of eagle prey and therefore reduce foraging opportunities for eagles; and/or deep snow may reduce the forage availability for ungulates such that they shift to roads, which increases collision risk and roadkill availability for golden eagles (Cunningham et al. 2022; Harlow and Menkens Jr. 1986; Riginos et al. 2016; Rogowitz 1997; Smith 1990; Steenhof et al. 1997). Accordingly, we predicted that the selection of areas near roads would increase with snow depth within an individual eagle's home range. To test this prediction, we calculated the average snow depth across all randomly drawn available points (average of 6.6 per individual per day) within an AID‐winter's home range for each day the individual wintered in Wyoming. We then assigned these values to both the used and available points for each individual each day. We opted for this approach as we were more interested in whether higher snow depths (within the home‐range) were associated with golden eagles selecting areas near roads, and not the depth of snow preferred by eagles. We derived snow depth from the Snow Data Assimilation System at a resolution of 1 × 1 km from 2014 to 2023 (National Operational Hydrologic Remote Sensing Center (NOHRSC) 2004). We sorted snow depth into five categorical groups, with each group defined based on the Interquartile Range of snow depth values derived from the available points. We used this method to bin snow depth categories because the distribution of snow depths was highly right skewed (i.e., a few very large snow depth values), and we wanted to assess variation in smaller snow depth values and moderate the influence of the very large snow depth values. Extracted snow depth values were classified as: “no snow” (0 cm), “low snow” (values > 0–2.3 cm), “moderate snow” (2.3–7.6 cm), “moderately high snow” (7.6–17.9 cm), and “high snow” (≥ 17.9 cm).

Our third extrinsic hypothesis (H7: prey energetic constraints) was that as winter progresses, prey often have fewer food resources available on the landscape and may display less anti‐predator behavior in exchange for food acquisition, making them more vulnerable and easier to obtain by predators (Oates et al. 2019). Accordingly, we predicted road use by golden eagles to decrease as winter progressed. Our fourth extrinsic hypothesis (H8: diminishing prey availability) was that golden eagles may experience diminishing availability of prey as winter progresses because of weather‐related mortality and lack of reproduction. Golden eagles may therefore turn to alternative food sources such as roadkill during winter (Bedrosian et al. 2017), and accordingly, we predicted roadway use by eagles to increase as winter progressed.

Finally, we hypothesized that golden eagles would turn to roadkill during years in which the abundance of primary live prey was lower (H9: primary prey availability). We indexed leporid availability in each winter using annual cottontail harvest data (from hunting surveys in Wyoming; Wyoming Game and Fish (WGFD) 2022). For the used and available points in each winter, we assigned the previous year's annual harvest values. We did this to align our definition of “biological year” relative to our movement data with how the Wyoming Game and Fish Department defined a “survey year” for annual cottontail harvest. For example, we defined a biological year (winter) as November 1st from a given year (*y*) to March 31st of (*y* + 1); whereas cottontail harvest surveys in the previous year were defined as June 1st of year (*y*) to May 31st of (*y* + 1).

### 2.5 Statistics and Model Selection

We fit our RSFs using generalized linear mixed models (GLMMs) with a logit link using the package “glmmTMB” in Program R version 4.3.0 (R Core Team 2023; Brooks et al. 2017). We accounted for individual variation in habitat selection by including animal ID‐year as a random intercept (Gillies et al. 2006). After fitting each model, we checked for spatial autocorrelation in the model residuals using Moran's *I*, an index used to determine whether predicted values of the model are independent based on the distance between data points (Dormann et al. 2007). As all our models yielded significant spatial autocorrelation (Moran's *I* > 0.5, Table S1), we added a distance‐weighted spatial autocovariate to each model and refit the model. We calculated the autocovariate using *k*‐nearest neighbor (*k* = 8) averages of the response variable using the ‘lag.listw()’ function in the spdep package in R (Tables 1 and 2, Table S1; Augustin et al. 1996; Dormann et al. 2007; Tikkanen et al. 2018).

Once we took into account spatial autocorrelation in our models (Table S1), we used Akaike's Information Criterion adjusted for small sample sizes (AIC_c_) to assess relative empirical support among the intrinsic and extrinsic hypotheses (Burnham and Anderson 2004). To determine the relative importance of a given extrinsic variable used in our suite of extrinsic hypotheses, we assessed whether a given extrinsic variable yielded an AIC ranking of ≥ 2 Δ AIC_c_ units less when compared to the top intrinsic model (Burnham and Anderson 2004). Once we identified the top model, we assessed the directionality of how variables influenced selection for areas near roads using a combination of plots of predicted relationships and interpretation of the direction of the estimated beta coefficients. Beta coefficients in an RSF analysis can be interpreted as follows: for a one‐unit increase in a beta estimate of a given variable, the relative log odds of use for that variable go up or down (depending on the direction of the beta coefficient). Lastly, we assessed how well our top model (including both intrinsic variables and factors associated with the top extrinsic hypothesis) performed overall by quantifying Spearman's Rank Correlation Coefficient (*r*_s_) averaged from *K*‐fold Cross Validation across *k* = 10 folds (Boyce et al. 2002).

## 3 Results

**FIGURE 2:**
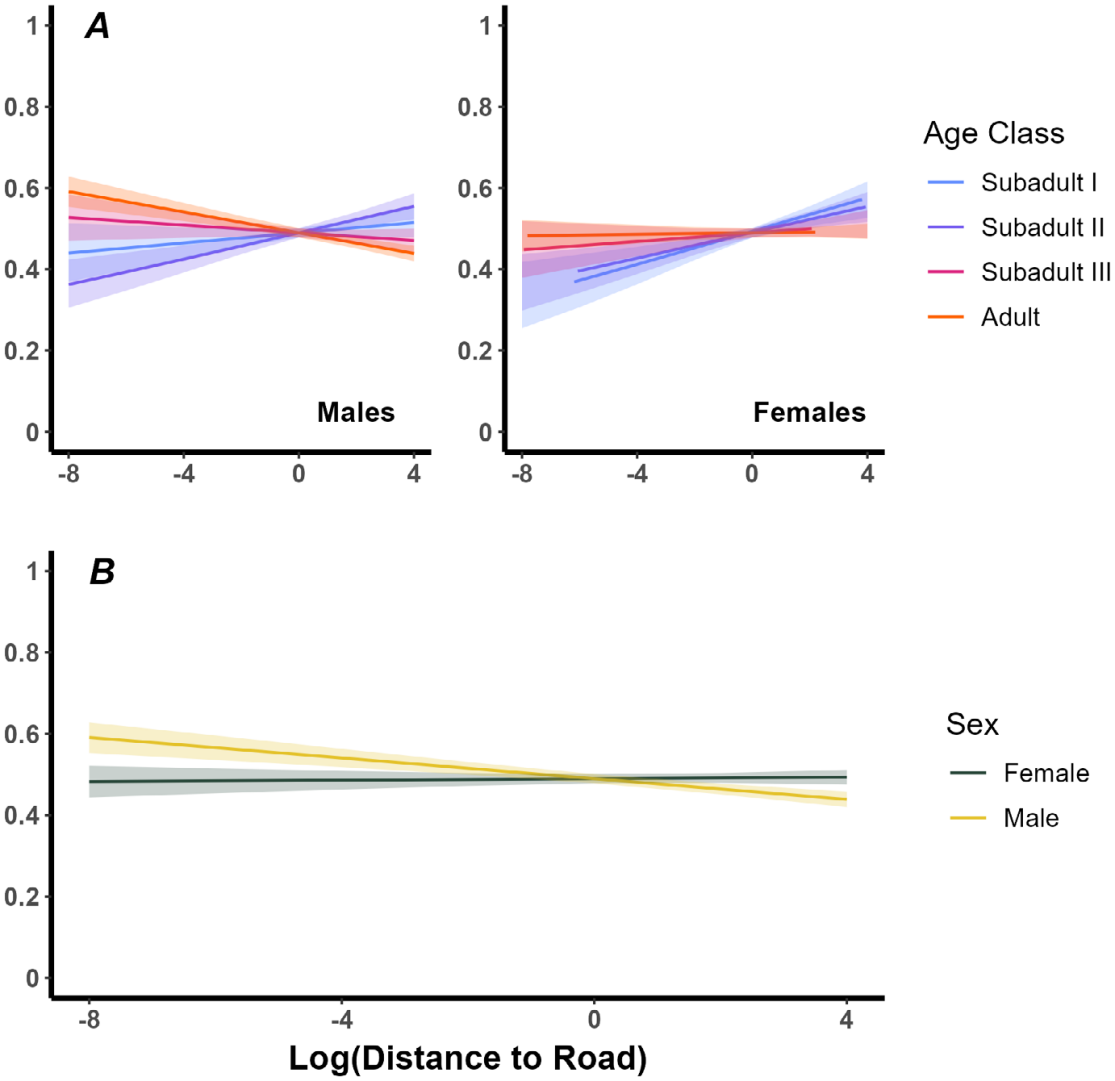
Relative probability of use of roadways by over‐wintering golden eagles (*n* = 51 individuals, 175 unique AID‐winters) as assayed by the distance to roads (Log scale) within golden eagle home ranges during 2013–2023. Model predictions derived from the sex & age dependent variation model (H3). Responses are displayed by focal intrinsic factors including age class and sex (A), sex (B), and movement strategy (migrants vs. year‐round residents; C). Shaded areas surrounding lines represent 95% CI intervals.

Without the inclusion of any intrinsic or extrinsic factors and accounting for spatial autocorrelation, golden eagles used areas near roads in accordance with their availability (*H*_base_, *β* = 0.001, *p* = 0.846; Table S2). Sex and age influenced selection for distance to roads (H3: *ω*_*i*_ = 0.6; AIC_c_ = 248,247.2 Tables S3–S4), whereas movement strategy did not (*H*_Global Intrinsic_: *ω*_*i*=_ 0.4; AIC_c_ = 248,248.0; Tables S3–S5 and S11–S13). Males were 14.2 times more likely to select areas near roads than females (*β*_males_ = −0.055; Table S4; Figure 2a,b). Adults, particularly adult males, selected areas near roads to a greater extent than any other age class (*β* = −2.71; Table S4; Figure 2a). Adult eagles were 16.3, 16, and 15.3 times more likely to select areas near roads compared with their subadult counterparts (Subadult I–III), respectively.

**FIGURE 3:**
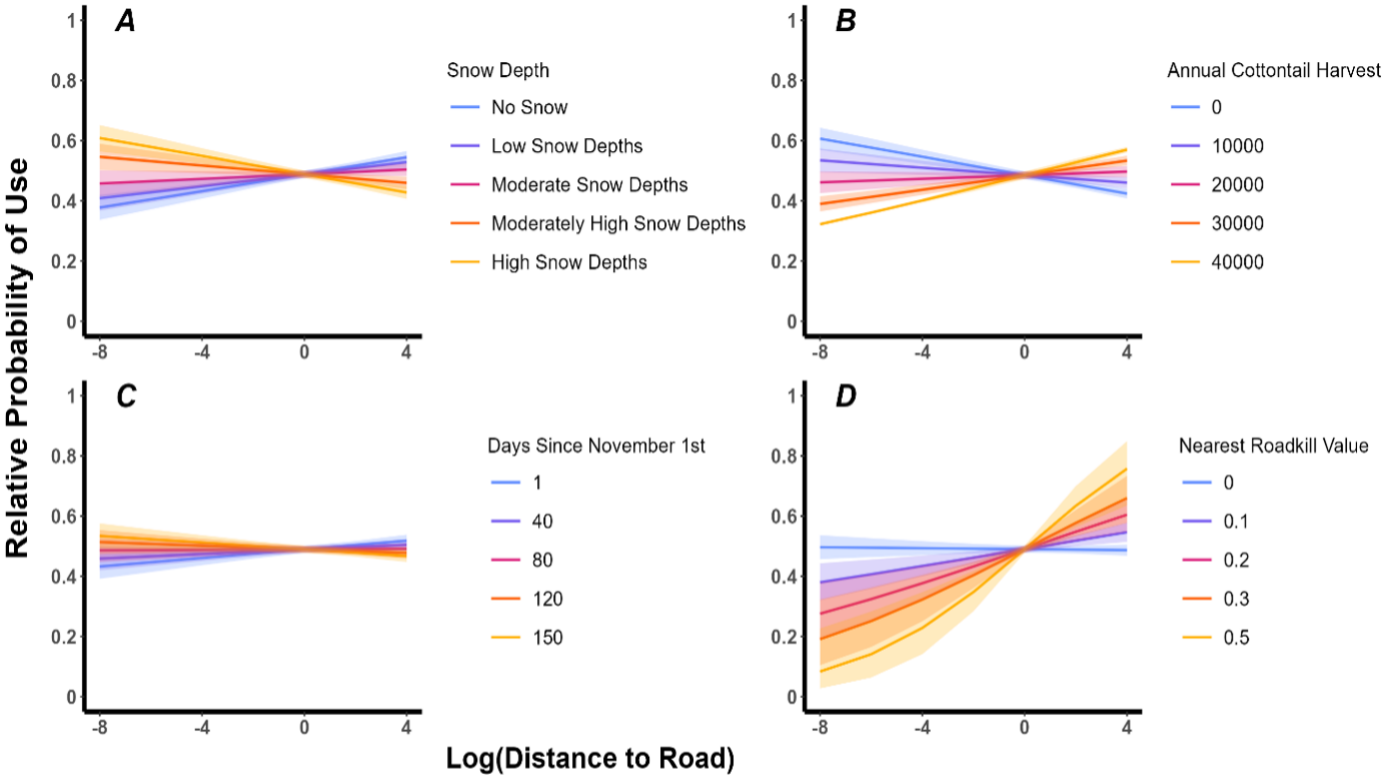
Relative probability of use (*y*‐axis) of roadways by over‐wintering golden eagles as assayed by the distance to roads (Log scale; *x*‐axis) within home ranges. Distance to road increases left to right along the *x*‐axis. Model predictions derived from each extrinsic model (H5–H9), *n* = 51 individuals, 175 unique AID‐winters) from 2013 to 2023. Responses are displayed by pertinent extrinsic factors, including snow depth (A), the relative availability of primary prey (B), the period of winter (C), and the nearest roadkill density (D). Shaded areas surrounding lines represent 95% CI intervals.

After accounting for intrinsic factors, the prey constraint (snow cover) hypothesis (H6) had the most empirical support for influencing eagle use of areas near roads during winter (AIC_c_ = 248,020.8; *ω*_*i*_ = 1.0; Tables S6 and S7). Golden eagles were more likely to select areas near roads when snow was deeper (Table S7; Figure 3a). During periods with high snow (> 17.9 cm), golden eagles were 17 times more likely to select areas near roads compared with periods of no snow (Table S7, Figure 3a). H6 had exceptional performance in predicting space use overall; *r*_s_ = 0.988. Although not as important as the influence of snow depth, each extrinsic model scored > 2 Δ AIC_c_ units lower than our top intrinsic model (H3: sex and age dependent variation); thus, explaining some more variation in habitat selection than the top intrinsic model (Tables S3 and S6). Specifically, golden eagles also tended to select areas closer to roads when the relative availability of their primary prey was low (Table S8; Figure 3b), during later periods in winter (Table S9; Figure 3c), and in areas where roadkill densities were low (Table S10; Figure 3d).

## 4 Discussion

Intrinsic and extrinsic factors can shape the use of space by animals in the wild. Risk–reward structures can differ within human‐altered landscapes, thereby necessitating an understanding of how animals can and will respond to anthropogenic change (Sih et al. 2011). In our study, both intrinsic and extrinsic factors influenced the selection of areas near roads by golden eagles in Wyoming during the winter. Adults and/or males were more likely to select habitats closer to roads within their home ranges compared with younger and/or female counterparts. After accounting for those intrinsic variables, snow depth overwhelmingly explained additional variation in the selection of areas near roads, where selection for areas near roads was higher when snow was deeper. Had we not accounted for both intrinsic and extrinsic factors, our conclusions would have been incomplete. For instance, our baseline model suggested that golden eagles do not select or avoid roads. Including only distance to roads in our resource selection function would have overlooked the strong variation in selection for roads among individuals and times of the year and years when snow depth was greater. Concomitant assessment of biologically relevant intrinsic and extrinsic factors can therefore provide a more nuanced and useful understanding of the contexts under which species of conservation concern tend to make potentially harmful spatial and habitat choices.

Variation in response to elements of risk is often attributed to differences in intrinsic traits such as the sex and age of an individual. Indeed, males and females tend to navigate risk differently (Stankowich 2008), potentially in association with variation in reproductive investment or body size (Corti and Shackleton 2002; Saïd et al. 2012). In our study, female golden eagles were less likely to select for roadways compared with males (Figure 2a,b; Table S4). We suspect that older females, particularly those of viable breeding age, may prefer habitats with ample abundance of primary prey (Collopy and Edwards 1989; Marzluff, Knick, et al. 1997; Wallace et al. 2024) where road density may be lower (Fensome and Mathews 2016) or farther away from roads (i.e., risk). Prey availability often is a primary factor influencing home range size in raptors (Marzluff, Kimsey, et al. 1997) and female golden eagles tend to have smaller home ranges compared with males during winter (Miller et al. 2017). Such differences may suggest that females are less likely to encounter roads during winter. Future research investigating the interplay between habitat quality and risk factors (e.g., roads) could reveal whether female golden eagles adopt a low‐risk reproductive investment strategy during the non‐breeding period (Reynolds et al. 2019). Such insights are important for understanding population viability across the full annual cycle and in response to habitat change (Marra et al. 2015).

Long‐lived animals typically have slower life‐history strategies with prolonged pre‐breeding stages and low reproductive potential (Reznick et al. 2002). This leaves species like the golden eagle highly susceptible to even seemingly small amounts of increased mortality, which can influence population dynamics (Sergio et al. 2021). Such populations can also be particularly sensitive to the mortality of older (breeding) individuals. We found that older birds were more likely to select areas near roads compared with younger birds, which is concerning from a population and conservation standpoint given the ubiquity of roads worldwide. Juvenile golden eagles (< 1 year old) in the western USA generally had lower survival rates than their older counterparts; however, older birds (> 1 year old) predominantly perished from anthropogenic sources of mortality (Millsap et al. 2022). Conversely, subadult golden eagles wintering in the Great Basin Desert tend to use habitats closer to roads (Hixson et al. 2022). Such differences could arise because of differing selective pressures (e.g., prey‐availability) across contexts.

Animals often adjust foraging behaviors based on current conditions within their environment. We found that golden eagles increased selection for areas near roads when snow depth was high, supporting our Prey Constraint (Snow Cover) Hypothesis. Higher snow depths can hamper locomotion and activity of prey (e.g., leporids; Kline 1965; Rogowitz 1997; Smith 1990), eagle foraging efficiency (Steenhof et al. 1997), and/or reduce the availability of forage for ungulates such that they shift to roads, thereby increasing collision risk and roadkill availability for golden eagles (Cunningham et al. 2022; Riginos et al. 2016). Such switching of foraging behavior by golden eagles is congruent with classic predictions of foraging theory within the context of risks given rewards (Brown and Kotler 2004). Similarly, golden eagles increased their use of wind farms, where mortality risk was higher, when prey densities in those areas were relatively high (Hunt 2002). Overall, our results generally support recent efforts to conceptually connect road ecology with predator–prey theory (Poulin et al. 2023).

Whereas support for most of our extrinsic hypotheses was limited compared with our prey constraint (snow cover) hypothesis (H6; Table S6), the roadkill availability (H5), prey energetic constraints (H7), diminishing prey availability (H8), and primary prey availability (H9) hypotheses all were > 2 AIC_c_ units better than our top intrinsic hypothesis (H3; Table S6). Because the RSF framework quantifies relative selection, we used AIC, which is also a relative assessment, to rank models (Burnham and Anderson 2004), and all of our variables were on different scales; the exact contribution (i.e., strength of each biological effect) of each of our hypotheses was difficult to quantify. Our results from the Roadkill Availability Hypothesis suggested that golden tended to select for areas near roads where roadkill densities were lower (Table S10; Figure 3d). This finding is likely associated with roadkill densities being strongly associated with higher traffic volumes (Riginos et al. 2016). Therefore, golden eagles could be targeting areas where foraging on roadkill during higher periods of snow has a good cost–benefit ratio (i.e., lower traffic volumes/risk of collision is lower; Slater et al. 2022). Results from our model structure representing Prey Energetic Constraints and the Diminishing Prey Availability hypotheses indicated an increase in selection for areas closer to roads as winter progressed, thus supporting the Diminishing Prey Availability Hypothesis (Table S9; Figure 3c). Such a finding could be a response to a reduction in live prey as leporids tend to experience high rates of mortality during winter (Gross et al. 1974; Hayden 1966), where eagles seek alternative prey resources such as roadkill (Bedrosian et al. 2017). Lastly, results from our model structure representing the Primary Prey Availability Hypothesis indicated that golden eagles select for areas closer to roads during periods of lower resource availability (Table S8; Figure 3b) which aligns well with results from our top model, whereby golden eagles select areas near roads when prey availability is constrained either by snow (see above) or by abundance.

Multiple caveats should be considered when interpreting our results. First, we did not use direct indices of prey availability. Rather, our index of prey availability was based on cottontail harvest during the previous year at a coarse (statewide) spatial resolution. While not available in Wyoming, direct measures of prey abundance and availability (e.g., Smith and Nydegger 1985) across years and with finer spatial resolution would greatly inform the mechanisms underlying the patterns of eagle space use we observed. Second, our metrics of prey vulnerability were based on time since winter started and snow depth. Other factors that we did not assess may also have influenced eagle habitat use and movements. For example, body condition can play a significant role in risk‐reward dynamics (Moran et al. 2021). Bioaccumulation of lead (both within season and with age) from foraging on carcasses can lead to poorer flight performance in golden eagles (Ecke et al. 2017), which might induce prioritization of easier prey acquisition over risk. Finally, whereas our inference is based on rather broad‐scale use of roads, finer‐scale observations of eagle behavior near roads, such as flight initiation distances or time spent on carcasses in relation to distance to roads, would greatly complement and enhance our findings (e.g., Slater et al. 2022).

Our results have important conservation implications, particularly because golden eagle visitation rates to roadkill during winter were highest in Wyoming compared with other U.S. states (Slater et al. 2022) and the risk of direct mortality to eagles from vehicle strikes (Millsap et al. 2022). The legal requirement of the Bald and Golden Eagle Protection Act is for no net loss of populations and, therefore, the goal of compensatory mitigation is to reduce the potential for negative population effects in areas with increased human‐associated risk of mortality such as those with roads or wind turbines (United States Fish and Wildlife Service (USFWS) 2024). Recent data in Wyoming have indicated a 28% population decline for the local population of golden eagles (Wyoming Game and Fish (WGFD) 2025). We found that specific components of the population (adults and males) selected for areas near roads to a greater extent than younger birds and females, which has implications for population‐level effects. Second, recent updates to the Rules and Regulations (50 CRF Parts 13 and 22) of “Permits for Incidental Take of Eagles and Eagle Nests” indicate that the option for using roadkill removal as mitigation is a work in progress (USFWS 2024). Our results underscore the potential conservation benefits, particularly for viable breeders (i.e., adults), of using roadkill removals as a mitigation approach, particularly during periods when snow is deep. Conversely, other techniques, such as the retrofitting of powerlines, typically disproportionately reduce mortality of younger birds (Mojica et al. 2018). Finally, our results can help inform the contexts (periods with deep snow, and to a lesser extent, later in winter and in winters after lower leporid harvest) under which investing in the removal of carcasses from roads would be most effective for limiting golden eagle mortality (Lonsdorf et al. 2018; Slater et al. 2022). Such considerations could greatly enhance mitigation geared towards populations of golden eagles, particularly within high‐quality breeding and over‐wintering areas (Dunk et al. 2019; Wallace et al. 2024).

## Author Contributions

**Joshua F. Layfield:** conceptualization (equal), formal analysis (equal), funding acquisition (supporting), investigation (lead), methodology (equal), software (equal), visualization (equal), writing – original draft (lead), writing – review and editing (equal). **Bryan Bedrosian:** conceptualization (equal), data curation (lead), funding acquisition (equal), investigation (lead), project administration (equal), resources (supporting), supervision (equal), validation (equal), visualization (supporting), writing – review and editing (equal). **Anna Chalfoun:** conceptualization (equal), investigation (supporting), project administration (equal), supervision (equal), validation (equal), visualization (supporting), writing – review and editing (equal). **Robert Domenech:** data curation (supporting), resources (supporting), writing – review and editing (supporting). **Stephen B. Lewis:** data curation (supporting), resources (supporting), writing – review and editing (supporting). **Brian W. Smith:** data curation (supporting), resources (supporting), writing – review and editing (supporting). **Jerod A. Merkle:** conceptualization (equal), data curation (supporting), formal analysis (equal), funding acquisition (equal), investigation (supporting), project administration (equal), resources (equal), software (equal), supervision (equal), validation (equal), visualization (equal), writing – review and editing (equal).

## Disclosure

Disclaimers: The USFWS relies on terms and conditions of its permits and those of the USGS, which USFWS employees are bound to adhere to, to ensure humane and ethical treatment of study animals. Findings and conclusions in this article are those of the authors and do not necessarily represent the views of the USFWS. Any use of trade, product, website, or firm names in this publication is for descriptive purposes only and does not imply endorsement by the US Government.

## Conflicts of Interest

The authors declare no conflicts of interest.

## Supporting information


**Data S1:** ece371955‐sup‐0001‐TableS1‐S13.docx.

## Data Availability

All the required data have been uploaded for review, but not for publication. Due to the sensitivity of the species investigated in this manuscript (the number one cause of anthropogenic mortality being direct persecution), we cannot make the raw data publicly available.
