# Supplementary material for: Intrinsic and Extrinsic Factors Simultaneously Modulated the Use of Roadways by Golden Eagles During Winter
Source: Ecol Evol. 2025 Aug 14;15(8):e71955. doi: 10.1002/ece3.71955 (PMC12353876; doi:10.1002/ece3.71955)
Supplement: Supplementary file 1 — Data S1: ece371955‐sup‐0001‐TableS1‐S13.docx. [file ECE3-15-e71955-s001.docx]

**Table S1**: Estimated parameters for Moran’s I across fitted intrinsic and extrinsic models. (Model Status) represents models tested for spatial autocorrelation with/without the spatial autocovariate (Hypothesis) represents the associated intrinsic or extrinsic model (Moran’s I) represents the degree of spatial autocorrelation detected, where values range from -1 to 1, with values near 0 represent no detection of spatial autocorrelation (P-value) represents the significance of the detected spatial autocorrelation

|  |  |  |  |
| --- | --- | --- | --- |
| **Model Status** | **Hypothesis** | **Moran’s I** | **P** |
| Models without Autocov | H_base_: Just Distance to Roads | 0.515 | < 0.001 |
|  | H1: Sex-Dependent Variation | 0.513 | < 0.001 |
|  | H2: Age-Dependent Variation | 0.513 | < 0.001 |
|  | H3: Sex - & Age Dependent Variation | 0.510 | < 0.001 |
|  | H4: Movement Strategy | 0.515 | < 0.001 |
|  | H_Global Intrinsic_: Global Intrinsic Model | 0.510 | < 0.001 |
|  | H5: Roadkill Availability | 0.509 | < 0.001 |
|  | H6: Prey Constrain (Snow Cover) | 0.510 | < 0.001 |
|  | H7 & H8: Prey Energetic Constraints/Diminishing Prey Availability | 0.510 | < 0.001 |
|  | H9: Primary Prey Availability | 0.508 | < 0.001 |
| Models with Autocov |  |  |  |
|  | H_base_: Just Distance to Roads | -0.091 | 1.00 |
|  | H1: Sex-Dependent Variation | -0.091 | 1.00 |
|  | H2: Age-Dependent Variation | -0.091 | 1.00 |
|  | H3: Sex - & Age Dependent Variation | -0.091 | 1.00 |
|  | H4: Movement Strategy | -0.091 | 1.00 |
|  | H_Global Intrinsic_: Global Intrinsic Model | -0.091 | 1.00 |
|  | H5: Roadkill Availability | -0.090 | 1.00 |
|  | H6: Prey Constraint (Snow) | -0.090 | 1.00 |
|  | H7 & H8: Prey Energetic Constraints/Diminishing Prey Availability | -0.091 | 1.00 |
|  | H9: Primary Prey Availability | -0.090 | 1.00 |

**Table S2**: Estimated statistical parameters of the base model H_base_. (β) represents the beta coefficients produced from the fixed effects included in the model. (SE) represents standard error estimates on β. (Z-value) represents the parameter divided by the SE. (P-value) represents the significance level in the model.

| **Hypothesis** | **Parameter** | **β** | **SE** | **Z** | **P** |
| --- | --- | --- | --- | --- | --- |
| H_Base_ | Intercept | -2.71 | 2.42E-02 | -111.8 | <0.001 |
|  | log(D2R) | 9.60E-04 | 4.94E-03 | 0.19 | 0.85 |
|  | Autocov | 5.05 | 1.66E-02 | 304.24 | <0.001 |

| **Table S3:** Statistical parameters from the top supported generalized linear mixed-effect models for habitat selection to areas near roads by golden eagles in Wyoming, USA from 2014–2023 from the intrinsic model suite. Models ranked by the number of model parameters (K), the corrected AIC scores (AICc), Akaike weights (ω_i_), and the log-likelihood (LL). | | | | | | |
| --- | --- | --- | --- | --- | --- | --- |
| **Hypothesis** | **Model Name** | **K** | **AICc** | **Δ AICc** | **ω_i_** | **LL** |
| H3 | Sex & Age Dependent Variation | 11 | 248247.2 | 0 | 0.6 | -124112.6 |
| H_Global Intrinsic_ | Global Intrinsic Model | 12 | 248248.0 | 0.78 | 0.4 | -124112.0 |
| H2 | Age Dependent Variation | 7 | 248265.9 | 18.75 | 0 | -124126.0 |
| H1 | Sex Dependent Variation | 5 | 248299.8 | 52.6 | 0 | -124144.9 |
| H_Base_ | Just Distance to Roads | 4 | 248314.1 | 66.97 | 0 | -124153.1 |
| H4 | Movement Strategy | 5 | 248316.0 | 68.86 | 0 | -124153.0 |

**Table S4:** Estimated statistical parameters of the top intrinsic model H3: Sex & Age Dependent Variation at the home range scale across golden eagles wintering in Wyoming from 2014–2023. (β) represents the beta coefficients produced from the fixed effects included in the model. (SE) represents standard error estimates on β. (Z-value) represents the parameter divided by the SE. (P-value) represents the significance level in the model.

| **Hypothesis** | **Parameter** | **β** | **SE** | **Z** | | **P** |
| --- | --- | --- | --- | --- | --- | --- |
| H3 | Intercept | -2.71 | 2.48E-02 | -109.2 | <0.001 | |
|  | log(D2R) | 3.62E-03 | 8.80E-03 | 0.41 | 0.68 | |
|  | Autocov | 5.04 | 1.66E-02 | 303.4 | < 0.001 | |
|  | log(D2R):Sex (Male) | -5.48E-02 | 1.23E-02 | -4.45 | < 0.001 | |
|  | log(D2R):Age (Subadult I) | 7.88E-02 | 2.45E-02 | 3.21 | 0.001 | |
|  | log(D2R):Age (Subadult II) | 6.04E-02 | 2.03E-02 | 2.97 | 0.003 | |
|  | log(D2R):Age (Subadult III) | 1.73E-02 | 1.91E-02 | 0.91 | 0.36 | |
|  | log(D2R):Sex (Male):Age (Subadult I) | -2.54E-03 | 3.18E-02 | -0.08 | 0.93 | |
|  | log(D2R):Sex (Male):Age (Subadult II) | 5.61E-02 | 2.72E-02 | 2.06 | 0.04 | |
|  | log(D2R):Sex (Male):Age (Subadult III) | 1.50E-02 | 2.53E-02 | 0.59 | 0.55 | |

| **Table S5:** Estimated statistical parameters of the Global intrinsic model H_Globabl Intrinsic_. (β) represents the beta coefficients produced from the fixed effects included in the model. (SE) represents standard error estimates on β. (Z-value) represents the parameter divided by the SE. (P-value) represents the significance level in the model. | | | | | |
| --- | --- | --- | --- | --- | --- |
|  |  |  |  |  |  |
|  |  |  |  |  |  |
|  |  |  |  |  |  |
| **Hypothesis** | **Parameter** | **β** | **SE** | **Z** | **P** |
| H_Global Intrinsic_ | Intercept | -2.71 | 2.50E-02 | -108.9 | < 0.001 |
|  | log(D2R) | 1.36 E-02 | 1.26E-02 | 1.08 | 0.28 |
|  | Autocov | 5.04 | 1.66E-02 | 303.4 | < 0.001 |
|  | log(D2R):Movement_Strategy (Resident) | -1.35E-02 | 1.22E-02 | -1.10 | 0.27 |
|  | log(D2R):Sex (Male) | -5.47E-02 | 1.23E-02 | -4.44 | < 0.001 |
|  | log(D2R):Age (Subadult I) | 8.26E-02 | 2.47E-02 | 3.34 | < 0.001 |
|  | log(D2R):Age (Subadult II) | 6.27 E-02 | 2.04 E-02 | 3.07 | < 0.001 |
|  | log(D2R):Age (Subadult III) | 1.96 E-02 | 1.92 E-02 | 1.02 | 0.31 |
|  | log(D2R):Sex (Male):Age (Subadult I) | -4.89 E-03 | 3.18 E-02 | -0.15 | 0.878 |
|  | log(D2R):Sex (Male):Age (Subadult II) | 5.65 E-02 | 2.73 E-02 | 2.07 | 0.04 |
|  | log(D2R):Sex(Male):Age (Subadult III) | 1.52 E-02 | 2.53 E-02 | 0.6 | 0.55 |

| **Table S6:** Statistical parameters from the top supported generalized linear mixed-effect models for habitat selection to areas near roads by golden eagles from 2014–2023 from the extrinsic model suite. Models ranked by the number of model parameters (K), the corrected AIC scores (AICc), difference in correct AIC scores between model (Δ AIC*_c_* ) Akaike weights (ωi), and the log-likelihood (LL). | | | | | | |  |  |
| --- | --- | --- | --- | --- | --- | --- | --- | --- |
|  |  |  |  |  |  |  |  |  |
|  |  |  |  |  |  |  |  |  |
|  |  |  |  |  |  |  |  |  |
|  |  |  |  |  |  |  |  |  |
|  |  |  |  |  |  |  |  |  |
| **Hypothesis** | **Model Name** | **K** | **AICc** | **Δ AICc** | **ω_i_** | **LL** |  |  |
| H6 | Prey Constraint (Snow Cover) | 15 | 248020.8 | 0 | 1 | -123995.4 |  |  |
| H9 | Primary Prey Availability | 12 | 248112.9 | 92.12 | 0 | -124044.5 |  |  |
| H7&H8 | Prey Energetic Constraints & Diminishing Prey Availability | 12 | 248207.5 | 186.71 | 0 | -124091.8 |  |  |
| H5 | Roadkill Availability | 12 | 248233.6 | 212.76 | 0 | -124104.8 |  |  |
| H3 | Sex- & Age Dependent Variation | 11 | 248247.2 | 226.37 | 0 | -124112.6 |  |  |

|  |
| --- |
|  |
|  |

**Table S7:** Estimated statistical parameters of the extrinsic model H6: Prey Constraint (Snow Cover) at the home range scale across golden eagles wintering in Wyoming from 2014–2023. (β) represents the beta coefficients produced from the fixed effects included in the model. (SE) represents standard error estimates on β. (Z-value) represents the parameter divided by the SE. (P-value) represents the significance level in the model.

| **Hypothesis** | **Parameter** | **β** | **SE** | **Z** | **P** |
| --- | --- | --- | --- | --- | --- |
| H6 | Intercept | -2.71 | 2.55E-02 | -106.2 | < 0.001 |
|  | log(D2R) | -6.10E-02 | 1.08E-02 | -5.67 | < 0.001 |
|  | Autocov | 5.04 | 1.66E-02 | 303.3 | < 0.001 |
|  | log(D2R):Sex (Male) | -6.27E-02 | 1.24E-02 | -5.04 | < 0.001 |
|  | log(D2R):Age (Subadult I) | 6.30E-02 | 2.48E-02 | 2.54 | 0.01 |
|  | log(D2R):Age (Subadult II) | 4.85E-02 | 2.05E-02 | 2.36 | 0.02 |
|  | log(D2R):Age (Subadult III) | 1.29E-02 | 1.93E-02 | 0.67 | 0.51 |
|  | log(D2R):Low_Snow_Depths | 1.01E-01 | 9.05E-03 | 11.2 | < 0.001 |
|  | log(D2R):Moderate_Snow_Depths | 7.65E-02 | 9.10E-03 | 8.41 | < 0.001 |
|  | log(D2R):Moderately_High_Snow_Depths | 3.20E-02 | 9.04E-03 | 3.55 | < 0.001 |
|  | log(D2R):No_Snow | 1.20E-01 | 9.08E-03 | 12.94 | < 0.001 |
|  | log(D2R):Sex (Male):Age (Subadult I) | 9.75E-03 | 3.20E-02 | 0.3 | 0.76 |
|  | log(D2R):Sex (Male):Age (Subadult II) | 7.10E-02 | 2.74E-02 | 2.58 | 0.009 |
|  | log(D2R):Sex(Male):Age (Subadult III) | 2.42 E-02 | 2.55E-02 | 0.95 | 0.34 |

**Table S8:** Estimated statistical parameters of the extrinsic model H9: Prey Availability (Cottontail) at the home range scale across golden eagles wintering in Wyoming from 2014–2023. (β) represents the beta coefficients produced from the fixed effects included in the model. (SE) represents standard error estimates on β. (Z-value) represents the parameter divided by the SE. (P-value) represents the significance level in the model.

| **Hypothesis** | **Parameter** | **β** | **SE** | **Z** | **P** |
| --- | --- | --- | --- | --- | --- |
| H9 | Intercept | -2.73 | 2.73E-02 | -99.72 | < 0.001 |
|  | log(D2R) | -6.16E-02 | 1.05E-02 | -5.86 | < 0.001 |
|  | Autocov | 5.03 | 1.66E-02 | 303.01 | < 0.001 |
|  | log(D2R):Sex (Male) | -5.18E-02 | 1.26E-02 | -4.13 | < 0.001 |
|  | log(D2R):Age (Subadult I) | -7.87E-02 | 2.85E-02 | -2.76 | 0.006 |
|  | log(D2R):Age (Subadult II) | -7.14E-02 | 2.37E-02 | -3.01 | 0.002 |
|  | log(D2R):Age (Subadult III) | -3.56E-02 | 2.01E-02 | -1.77 | 0.08 |
|  | log(D2R):Cottontail_Harvest | 3.67E-06 | 3.13E-07 | 11.74 | < 0.001 |
|  | log(D2R):Sex (Male):Age (Subadult I) | 3.82E-02 | 3.27E-02 | 1.17 | 0.24 |
|  | log(D2R):Sex (Male):Age (Subadult II) | 6.71E-02 | 2.78E-01 | 2.42 | 0.02 |
|  | log(D2R):Sex(Male):Age  (Subadult III) | -6.50E-03 | 2.59E-02 | -0.25 | 0.8 |

**Table S9:** Estimated statistical parameters of the extrinsic model H7&H8: Energetic Constraints Prey / Diminishing Prey Availability at the home range scale across golden eagles wintering in Wyoming from 2014–2023. (β) represents the beta coefficients produced from the fixed effects included in the model. (SE) represents standard error estimates on β. (Z-value) represents the parameter divided by the SE. (P-value) represents the significance level in the model.

| **Hypothesis** | **Parameter** | **β** | **SE** | **Z** | **P** |
| --- | --- | --- | --- | --- | --- |
| H7&8 | Intercept | -2.71 | 2.48E-02 | -108.9 | <0.001 |
|  | log(D2R) | 2.91E-02 | 9.62E-03 | 3.02 | 0.002 |
|  | Autocov | 5.04 | 1.66E-02 | 303.4 | < 0.001 |
|  | log(D2R):Sex (Male) | -5.51E-02 | 1.23E-02 | -4.47 | < 0.001 |
|  | log(D2R):Age (Subadult I) | 8.41E-02 | 2.45E-02 | 3.43 | < 0.001 |
|  | log(D2R):Age (Subadult II) | 6.23E-02 | 2.03E-02 | 3.06 | 0.002 |
|  | log(D2R):Age (Subadult III) | 1.79E-02 | 1.91E-02 | 0.94 | 0.35 |
|  | log(D2R):Time_Across_Winter | -3.42E-04 | 5.30E-05 | -6.45 | < 0.001 |
|  | log(D2R):Sex (Male):Age (Subadult I) | -4.85E-03 | 3.18E-02 | -0.15 | 0.88 |
|  | log(D2R):Sex (Male):Age (Subadult II) | 5.65E-02 | 2.75E-02 | 2.07 | 0.04 |
|  | log(D2R):Sex(Male):Age (Subadult III) | 1.62E-02 | 2.52E-02 | 0.64 | 0.52 |

**Table S10:** Estimated statistical parameters of the extrinsic model H5: Roadkill Availability at the home range scale across golden eagles wintering in Wyoming from 2014–2023. (β) represents the beta coefficients produced from the fixed effects included in the model. (SE) represents standard error estimates on β. (Z-value) represents the parameter divided by the SE. (P-value) represents the significance level in the model.

| **Hypothesis** | **Parameter** | **β** | **SE** | **Z** | **P** |
| --- | --- | --- | --- | --- | --- |
| H5 | Intercept | -2.71 | 2.49E-02 | -108.7 | < 0.001 |
|  | log(D2R) | -3.22E-03 | 8.96E-03 | -0.36 | 0.72 |
|  | Autocov | 5.04 | 1.66E-02 | 303.19 | < 0.001 |
|  | log(D2R):Sex (Male) | -5.86E-02 | 1.24E-02 | -4.73 | < 0.001 |
|  | log(D2R):Age (Subadult I) | 7.98E-02 | 2.46E-02 | 3.25 | 0.001 |
|  | log(D2R):Age (Subadult II) | 6.17E-02 | 2.04E-02 | 3.03 | 0.002 |
|  | log(D2R):Age (Subadult III) | 1.84E-02 | 1.12E-02 | 0.96 | 0.33 |
|  | log(D2R):Nearest_Roadkill_Density | 0.59 | 1.51E-01 | 3.94 | < 0.001 |
|  | log(D2R):Sex (Male):Age (Subadult I) | -2.99E-03 | 3.18E-02 | -0.09 | 0.92 |
|  | log(D2R):Sex (Male):Age (Subadult II) | 5.43E-02 | 2.73E-02 | 1.99 | 0.05 |
|  | log(D2R):Sex(Male):Age (Subadult III) | 1.63E-02 | 2.53E-02 | 0.64 | 0.52 |

**Table S11:** Estimated statistical parameters of the intrinsic model H1: Sex Dependent Variation at the home range scale across golden eagles wintering in Wyoming from 2014–2023. (β) represents the beta coefficients produced from the fixed effects included in the model. (SE) represents standard error estimates on β. (Z-value) represents the parameter divided by the SE. (P-value) represents the significance level in the model.

| **Hypothesis** | **Parameter** | **β** | **SE** | **Z** | **P** |
| --- | --- | --- | --- | --- | --- |
| H1 | Intercept | -2.72 | 2.47E-02 | -109.65 | <0.001 |
|  | log(D2R) | 2.31E-02 | 7.13E-03 | -3.01 | 0.003 |
|  | Autocov | 5.04 | 1.66E-02 | 303.95 | <0.001 |
|  | log(D2R):Age (Subadult I) | -3.76E-02 | 9.35E-03 | -4.03 | <0.001 |

**Table S12:** Estimated statistical parameters of the intrinsic model H2: Age Dependent Variation at the home range scale across golden eagles wintering in Wyoming from 2014–2023. (β) represents the beta coefficients produced from the fixed effects included in the model. (SE) represents standard error estimates on β. (Z-value) represents the parameter divided by the SE. (P-value) represents the significance level in the model.

| **Hypothesis** | **Parameter** | **β** | **SE** | **Z** | **P** |
| --- | --- | --- | --- | --- | --- |
| H2 | Intercept | -2.7 | 2.42E-02 | -111.5 | <0.001 |
|  | log(D2R) | -2.31E-02 | 6.37E-03 | -3.63 | <0.001 |
|  | Autocov | 5.04 | 1.66E-02 | 303.9 | <0.001 |
|  | log(D2R):Age (Subadult I) | 6.95E-02 | 1.54E-02 | 4.5 | <0.001 |
|  | log(D2R):Age (Subadult II) | 8.70E-02 | 1.34E-02 | 6.48 | <0.001 |
|  | log(D2R):Age (Subadult III) | 1.96E-02 | 1.23E-02 | 1.59 | 0.11 |

**Table S13:** Estimated statistical parameters of the intrinsic model H4: Movement Strategy at the home range scale across golden eagles wintering in Wyoming from 2014–2023. (β) represents the beta coefficients produced from the fixed effects included in the model. (SE) represents standard error estimates on β. (Z-value) represents the parameter divided by the SE. (P-value) represents the significance level in the model.

| **Hypothesis** | **Parameter** | **β** | **SE** | **Z** | **P** |
| --- | --- | --- | --- | --- | --- |
| H4 | Intercept | -2.71 | 2.43E-02 | -111.7 | <0.001 |
|  | log(D2R) | -2.24E-03 | 1.10E-02 | -0.21 | 0.837 |
|  | Autocov | 5.05 | 1.66E-02 | 304.23 | <0.001 |
|  | log(D2R):Movement_Strategy (Resident) | 3.90E-03 | 1.18E-02 | 0.33 | 0.741 |
